# Disparities in Prevalence of Smoking and Smoking Cessation during Pregnancy: A Population-Based Study

**DOI:** 10.1155/2015/345430

**Published:** 2015-05-14

**Authors:** Josiane L. Dias-Damé, Juraci A. Cesar

**Affiliations:** ^1^Programa de Pós-Graduação em Epidemiologia, Departamento de Medicina Social, Faculdade de Medicina, Universidade Federal de Pelotas, 96020-220 Pelotas, RS, Brazil; ^2^, Brazil Divisão de População & Saúde, Faculdade de Medicina, Universidade Federal do Rio Grande (FURG), 96200-400 Rio Grande, RS, Brazil

## Abstract

*Objective*. To examine time trends in prevalence of smoking and smoking cessation during pregnancy by family income, maternal level of education, skin color, and age. *Methods*. We conducted three population-based surveys in 2007, 2010, and 2013 with newly delivered mothers living in the municipality of Rio Grande, Southern Brazil. Data were collected using questionnaires administered after delivery in all (two) maternity units in the city, at Dr. Miguel Riet Corrêa Júnior Hospital and at Santa Casa de Misericórdia. Time trends were analyzed using chi-square test for linear trend. *Results*. Data of 7,572 women showed that the prevalence of smoking before pregnancy decreased from 28% (26.2–29.7) in 2007 to 22% (20.8–24.0) in 2013 (*P* < 0.001). Prevalence of smoking during pregnancy decreased from 22% (20.4–23.7) in 2007 to 18% (16.6–19.5) in 2013 (*P* < 0.001). This reduction varied across income ranging from 17% (poorest) to 35% (richest) (*P* < 0.001). The lower the income, the higher the smoking prevalence during pregnancy. Smoking cessation was more prevalent among women of higher level of education and income. *Conclusions*. Smoking before and during pregnancy is still highly prevalent and the prevalence of cessation is low pointing to a need to strengthen actions targeting low-income, less educated, black pregnant women.

## 1. Introduction

Smoking during pregnancy is recognized as one of the most important preventable risk factors for adverse pregnancy and birth outcomes. Smoking is associated with fetal growth restriction, stillbirth, premature delivery, premature rupture of membranes, and sudden infant death [1–4]. Besides increasing the risk of miscarriage, ectopic pregnancy, and placenta previa, smoking during pregnancy can also predispose the offspring to behavioral and cognitive disorders, overweight, obesity, respiratory diseases, reduced lung function, and tobacco addiction [2, 3, 5–7].

In addition to its direct effect on perinatal outcomes, smoking during pregnancy has a role as a potential mediator of socioeconomic differences in these outcomes [8–10]. It is strongly associated with socioeconomic condition [11], being more prevalent among less educated women [12–17] and those in the lowest income group [13, 18]. Most studies analyzing time trends in smoking during pregnancy have reported a decrease in prevalence in any period studied [15, 19–28]. Yet, the downward trend of smoking prevalence has varied by levels of socioeconomic condition among pregnant women, with smaller decreases among those in the lowest level [26, 28].

Smoking cessation during pregnancy is as expected higher among high-income pregnant women [20, 22, 25, 29]. However, studies of time trends in smoking cessation are scarce with inconsistent findings showing increase [20], decrease [25, 30], and stability of prevalence [22].

Time trends in prevalence of smoking cessation during pregnancy have been little investigated, and no studies could be found of trends in socioeconomic differences in smoking cessation during pregnancy. In addition, there is a scarcity of population-based studies of smoking and smoking cessation during pregnancy in Brazil.

This study aimed to examine the time trends in prevalence of smoking before and during pregnancy and in prevalence of smoking cessation during pregnancy in 2007, 2010, and 2013 by family income quintile, maternal level of education, maternal skin color, and maternal age.

## 2. Methods

A cross-sectional study is carried out in Rio Grande every three years to assess pregnancy and childbirth care among newly delivered women living in this municipality. Rio Grande is located in southern Brazil, about 300 km from Rio Grande do Sul state's capital Porto Alegre, and it has a population of about 210,000.

The present study was based on data collected in three surveys conducted in Rio Grande in 2007, 2010, and 2013. The target population was all women living in the municipality who gave birth in all (two) maternity units in the city (Dr. Miguel Riet Corrêa Júnior Hospital of the Universidade Federal do Rio Grande Medical School and Santa Casa de Misericórdia de Rio Grande) between January 1 and December 31 in each year studied. Given that over 99% of deliveries take place in the municipality's hospitals, these are census surveys; that is, they include all births taking place in the municipality during the study period. Newly delivered women of newborns born before the 20th week of gestation and/or weighing less than 500 g were excluded from the study.

All information here presented was collected using a standard questionnaire administered by trained interviewers. Interviewers attended a 40-hour training about a month before they began working on data collection each year of survey. The training sessions included questionnaire and instruction manual reading and simulation interviews. We carried out a pilot study in the maternity hospitals to be included in the study and tested the questionnaire and data collection logistics.

Female interviewers paid daily visits early in the morning to the municipality's two maternity hospitals. They checked admission records for any birth delivery that took place the day before and then double-checked this information against the hospital's Medical Statistics Service Database (SAME). When there was a record of a birth the day before, they made a note of the mother's name and proceeded to the postnatal ward. The interviewers approached the new mother and confirmed she lived either in the urban or in the rural area of Rio Grande. They then described the study and read a free and informed consent form and invited the mother to participate. Those who agreed signed two copies of the consent form and they were given one copy for their records. After the interview, questionnaires were coded and sent along with a copy of the signed consent form to the study site. All consent forms were filed at the study site and all questionnaire answers were reviewed and coded for data entry. The data were double-entered into Epidata 3.1 by different staff; they were entered in the second time in the reverse order. We conducted data entry checks for every set of 100 questionnaires and corrected any discrepancies. After completing data entry, a single file was created containing the data of all mothers and their newborns.

All information about smoking before and during pregnancy was collected in standard manner in all three surveys. “Smoking before pregnancy” was defined as having smoked at least one cigarette a day in the six months prior to the current pregnancy. “Smoking during pregnancy” was defined as smoking at least one cigarette a day every day during at least one trimester of pregnancy. “Smoking cessation during pregnancy” was defined as having smoked at least one cigarette a day in the six months prior to the current pregnancy and not smoking throughout the current pregnancy.

The exposure variables included maternal age (collected as a discrete variable and categorized into 13–19; 20–24; 25–29; 30 years or more); maternal self-referred skin color (white, mixed (an intermediate skin color between white and black, known as brown,* moreno* and/or mulatto), or black); maternal level of education (collected as a discrete variable and categorized into 0–4; 5–8; 9–11; and 12 full years or more); and monthly family income (in Brazilian reais, collected as a continuous variable and categorized into quintiles).

Fieldwork supervisors carried out quality control by performing phone interviews with a reduced sample comprising 10% of the participants. The kappa coefficient varied according to the characteristics studied and survey year. We calculated the coefficient of agreement for 24 questions and it ranged from 0.61 for “reason for cesarean section” to 0.92 for “type of delivery.” For most other questions, it was greater than 0.70, which is considered satisfactory. In addition, birth records at the maternity hospitals were examined by the interviewers on a daily basis and by the supervisors on a monthly basis.

We used descriptive statistics to calculate proportions and related 95% confidence intervals (95% CIs) for the variables studied. The chi-square test for linear trend in proportions was performed to examine time trends for each survey and for the entire study period. The chi-square test of heterogeneity was conducted to assess differences across categories within each survey as well as in percent decreases during the study period. All the statistical tests were two-sided, and the significance level was set at 5%. The analyses were performed with Stata 12 software (StataCorp, College Station, TX, USA).

The survey studies were approved by the Universidade Federal do Rio Grande Research Ethics Committee (CEPAS/FURG) and the A.C. Santa Casa do Rio Grande Heath Research Ethics Committee. All participants signed a free and informed consent form before the interviews. They were also informed about their right to decline participation and their right of confidentiality respect to any information obtained in the course of the research project.

## 3. Results

7,572 women were interviewed in the three surveys: 2,540 in 2007, 2,379 in 2010, and 2,653 in 2013. The rates of losses and refusals were 1.3% in 2007, 2.8% in 2010, and 2.3% in 2013 (Table 1). To calculate sample loss and refusals we used data from the National Live Birth Database (SINASC) as gold standard. SINASC gathers birth information by mother's place of residence from all Brazilian municipalities. Most mothers were 25 years or older (54%) and self-referred as white (67%) and had nine full years of schooling or more (56%).

Table 2 shows that overall prevalence of smoking before pregnancy fell from 27.9% in 2007 to 22% in 2013, a 19.7% decrease (*P* < 0.001). A downward trend was seen in smoking prevalence among women with high-income level (third and fourth quintiles) (*P* < 0.05), 9–11 years of schooling (*P* < 0.05), and white skin color (*P* < 0.001) and in almost all age groups (*P* ≤ 0.01) except for 25–29 years old. In all three surveys, smoking before pregnancy was generally more prevalent among women in the lowest income quintile (poorest), less educated and self-referred as black.

Table 3 shows that overall prevalence of smoking during pregnancy fell from 22% in 2007 to 18% in 2013, an 18% decrease (*P* < 0.001). In all three surveys, smoking was generally more prevalent among women in the lowest income quintile (poorest). When we examined smoking prevalence by family income between 2007 and 2013, we found that this decrease was not uniform across income quintiles: there was a 17% decrease in the lowest quintile while it was twice as much (35%) in the highest quintile (*P* < 0.001). The largest percent decreases were observed among women in the highest quintile (35%, *P* < 0.05), of white skin color (21.5%, *P* = 0.001) and younger (29.4%, *P* < 0.05).

Table 4 presents the prevalence of smoking cessation during pregnancy. There was a slight reduction (2%) during the study period, though not significant (*P* > 0.05). In all three surveys, smoking cessation was generally more prevalent among more educated women (12 years of schooling or more) and those who were in the highest income quintile (richest). Regarding level of education, in all three surveys, there was an increasing trend in smoking cessation as the level of education increased (*P* < 0.001). The same trend was observed in all three surveys by income quintiles as smoking cessation increased with family income.

## 4. Discussion

This study found a significant reduction in the prevalence of smoking before and during pregnancy between 2007 and 2013, which was more pronounced among younger women, of white skin color, in the highest income quintile. Smoking cessation during pregnancy was more prevalent among more educated women and those who were in the highest income quintile.

One of the limitations of this study lies in the fact that smoking was self-reported by the mothers. Despite the potential underestimation of smoking prevalence and the potential overestimation of smoking cessation during pregnancy, there is no reason to believe they occurred differently in the three surveys studied. Nevertheless, self-reported smoking status is the most widely used method for assessing smoking in population-based studies.

While self-reported smoking is recognized as an appropriate measure to assess exposure to tobacco during pregnancy [31, 32], some authors have argued it may underestimate the prevalence of smoking [33–35] and affect estimates of the effects of smoking [36]. Researchers have suggested biochemical markers for measuring tobacco exposure in pregnant women, but the prevalence of concealment of smoking status may vary depending on the choice of cutoff points [37]. The use of biochemical markers is costly and requires adequate cutoff points for pregnant women. Variations in nicotine metabolism need to be taken into account when setting up cutoff points for this specific population [38] and these cutoff points have to reliably differentiate intermittent from passive smokers [39]. Recent population-based studies conducted in Norway [31] and the US [40] showed that, among women who reported not smoking during pregnancy, only 2% showed cotinine levels consistent with active smoking.

Another limitation of this study is its cross-sectional design that may give rise to recall bias. In order to minimize recall bias, specific questions about smoking and smoking cessation were asked at different times between the six months before pregnancy until the last trimester. The objective was to attain a higher consistency in the response, as the interviewee answered several questions on the subject. We believe that this brought more reliability to the information collected, because all of the answers should have been consistent. This differs from asking a single isolated question about the issue, in which case it would not be possible to evaluate inconsistencies.

The operational definition of what constitutes smoking is a major concern in both public health and clinical practice settings. This issue is of particular importance in population-based studies because smoking and smoking cessation are characterized using low-cost straightforward approaches including few questions. However, there is a lack of a consistently applied definition of smoking and smoking cessation during pregnancy in epidemiological studies. In this study, we applied the definition of smoking during pregnancy as proposed by Santos et al., 2008 [28], that is, smoking at least one cigarette a day every day during at least one trimester of pregnancy. As for smoking cessation, we did not find any other study in the literature that used the same information we collected in our study (smoking status 6 months before pregnancy and smoking status at every trimester of pregnancy) in the operational definition of “smoking cessation.” Therefore, we developed the criteria presented in our manuscript. Yet, the different definitions of what constitutes smoking and smoking cessation during pregnancy must be taken into consideration when comparing studies.

In contrast to the reduction in smoking before pregnancy observed in our study, previous studies reported that the prevalence of smoking 3 months before pregnancy was stable at around 26% [20] and 22% [22]. The downward trend in prevalence observed among women with higher income (third and fourth quintiles), 9–11 years of schooling, and white skin color and in almost all age groups except for 25–29 years old suggests some women of reproductive age are quitting smoking to be prepared for pregnancy or even a reduction in the smoking initiation among those women. Similarly to that reported in previous studies, we found higher smoking prevalence before pregnancy among less educated women [20] and those who were in the lowest income quintile [18].

A reduction in the prevalence of smoking during pregnancy is consistent with that reported in most other studies [15, 19–23, 25–28] despite socioeconomic differences between countries. The 18% decrease in the prevalence during the study period corresponded to a 3% annual decrease and was similar to that observed (2.7%) in New South Wales, Australia, between 1994 and 2007 [26]. However, a study conducted in Maine, US, from 2000 to 2010, reported a 0.02% annual increase in the prevalence of women who smoked in the last trimester of pregnancy [41].

We found in our study that the downward trend in prevalence of smoking during pregnancy was significant only among women in the upper and lower income quintiles. However, similarly to that reported in previous studies [26, 28], it was not uniform across the categories studied, with marked reductions among high-income women (35% versus 17%). This finding conforms with the predictions of a model of the cigarette epidemic in developed countries that was proposed by Lopez et al. in 1994 [42]. According to this model, individuals of higher socioeconomic condition are more susceptible to the influence of mass media campaigns about the health risks of smoking. These results are confirmed by Santos et al. [28] who reported a decrease in the prevalence of smoking from 24.9% to 8.7% among high-income women compared to 43.7% to 33.6% among low-income women. Another study conducted in New South Wales found a 67.9% reduction in smoking prevalence among pregnant women of higher socioeconomic condition compared to just 25.9% among those of lower condition. In contrast to our results, these authors found further prevalence reductions among older women [26], but smoking during pregnancy was defined as “ever smoked during the current pregnancy.” The same was reported by Tong et al. [22] who found a significant decrease in smoking during the last trimester of pregnancy only among women of age 35 years or more.

Increasing trend in smoking prevalence as family income decreased is in agreement with some earlier studies conducted in Canada [13] and the United States [18], both based on secondary data. Although there was no significant trend in level of education, we found higher smoking prevalence among less educated women (0–4 years of schooling), as previously reported in other studies based on secondary [12–16] and primary data [17, 43].

Considering the harmful effects of smoking during pregnancy, pregnancy can be a favorable time to encourage smoking cessation since the possibility of harming their offspring may motivate smoking abstinence among pregnant women. Many women quit smoking as soon as they learn that they are pregnant, but those who continue to smoke are likely a subgroup that would be more resistant to change and thus require further investigation [44]. Pregnant women who quit smoking during pregnancy often have a profile that contrasts to that of those who continue to smoke. Those who quit are more likely to be primiparous women, of higher income, more educated, married, have a planned pregnancy and early prenatal care, and smoke fewer cigarettes per day [20, 22, 25, 45]. Our study found, in all three surveys, an increasing trend in the prevalence of smoking cessation with income and education increase. US studies conducted based on the Pregnancy Risk Assessment Monitoring System (PRAMS) surveillance data that defined smoking cessation as when women who smoked three months before pregnancy quit smoking by the last trimester reported a direct association between the prevalence of smoking cessation and education [20, 22].

In view of appeals and effort to raise awareness of the harmful health effects of smoking during pregnancy, we expected an increase in prevalence of smoking cessation during the study period. However, we found no other studies in Brazil that examined smoking cessation trends among pregnant women. Studies conducted in other countries have reported inconsistent results: upward [20], downward [25, 30], or even stable trends [22]. The results of this study may be due purely to chance because of very high *P* values (overall *P* value = 0.919). However, it may also suggest that governmental antismoking programs have been effective at preventing initiation of tobacco use but not at promoting smoking cessation. Brazil has established the National Tobacco Control Program comprising actions that promote smoke-free environments and smoking cessation projects. This program recommends that all pregnant women and nursing mothers have access to a cognitive-behavioral approach for smoking cessation. However, there are no interventions or programs specifically targeted to pregnant women.

It seems that current antismoking programs in Brazil have had varied effectiveness for preventing initiation of smoking and for smoking cessation during pregnancy. There is a need for different approaches. However, this should be monitored continually to acquire better evidence and, if necessary, to understand the causes of the decrease in smoking cessation in order to develop new actions for promoting smoking cessation during pregnancy.

The finding of an association between prevalence of smoking and smoking cessation with socioeconomic status (SE) over time could be explained by the Fundamental Cause theory. According to this theory, the SES is related to several risk factors, influences multiple outcomes, and involves access to resources that increase survival. Through new knowledge, this may lead to the establishment of preventive measures against risk factors, to the control of diseases, and to the prevention of diseases and associated complications [46]. However, this theory has some limitations including the fact that the resources for interventions are not always available at the time and in the required amount. Thus, the mechanisms that cause particular diseases change over time, the relationship between the SES and the occurrence of unfavorable outcomes can be weakened or even disappear, exposure can become irregular or unstable, and there may not be enough knowledge to propose new interventions.

Finally, considerable challenges remain to be tackled. In addition to those stated in the Fundamental Cause theory, the challenges for governments are to develop interventions, to reduce risk factors and prevent unfavorable health outcomes, to eliminate socioeconomic inequalities in health and access to health interventions, and to develop interventions of acceptable costs that are at its core more equally available among different population groups. Otherwise, this is likely to perpetuate the fact that the most affected are those from the lowest socioeconomic groups.

## 5. Conclusions

Despite the observed reduction, smoking before and during pregnancy is still highly prevalent among some women groups and prevalence of smoking cessation is low in almost all groups studied. There is a need to identify and provide treatment to female smokers before pregnancy or early in pregnancy during prenatal care. Moreover, it is necessary to strengthen ongoing actions and target low-income, less educated, black pregnant women.

## Figures and Tables

**Table 1:** Comparison of some indicators among the three surveys, 2007–2013. Perinatal study, Rio Grande, RS, Brazil.

Indicator	2007	2010	2013
Eligible newly delivered women	2,574	2,448	2,715
Nonresponse rate (%)	1.3	2.8	2.3
Final sample	2,540	2,379	2,653
Education (years), mean (SD^∗^)	8.6 (3.5)	9.0 (3.2)	9.5 (3.3)
Monthly family income (Brazilian reais), median	800	1,000	1,800
Age (years), mean (SD^∗^)	25.6 (6.6)	25.9 (6.4)	26.3 (6.5)
Self-referred skin color as white (%)	69.2	69.4	66.1
Prevalence of smoking before pregnancy (95% CI)	27.9 (26.2–29.7)	26.3 (24.5–28.1)	22.4 (20.8–24.0)
Prevalence of smoking during pregnancy (95% CI)	22.0 (20.4–23.7)	21.0 (19.3–22.6)	18.0 (16.6–19.5)
Prevalence of smoking cessation during pregnancy (95% CI)	18.0 (15.3–21.1)	21.1 (17.9–24.5)	17.6 (14.7–20.9)

^∗^SD: standard deviation.

**Table 2:** Prevalence of smoking 6 months before pregnancy by family income, level of education, skin color, and age. Rio Grande, RS, Brazil, 2007–2013 (*N* = 7,572).

Variable/survey year	2007		2010		2013		% change	*P* value for trend in the study period
	*n*	%	*n*	%	*n*	%	2007–2013	
Overall	2,540	27.9	2,379	26.3	2,653	22.4	−19.7	<0.001
Family income quintile								
1 (lowest)	580	41.7	539	32.1	622	33.8	−18.9	0.001^∗^
2	449	31.0	473	35.1	504	26.8	−13.5	0.019^∗^
3	449	27.9	435	28.3	469	22.0	−21.1	0.039
4	526	23.0	513	21.8	533	17.3	−24.8	0.021
5 (highest)	486	14.0	419	12.4	525	10.5	−25.0	0.088
*P* value for linear trend	<0.001		<0.001^∗^		<0.001			
Level of education (full years)								
0–4	320	39.4	190	43.7	162	38.9	−1.3	0.927
5–8	917	38.1	884	38.0	892	34.6	−9.2	0.134
9–11	1,064	20.2	1,060	17.1	1,188	16.6	−17.8	0.027
12 or more	239	7.9	245	10.6	411	6.3	−20.2	0.322
*P* value for heterogeneity	<0.001		<0.001		<0.001			
Self-referred skin color								
White	1,671	24.7	1,630	22.6	1,751	19.2	−22.3	<0.001
Mixed	517	31.9	507	34.7	593	28.2	−11.6	0.157
Black	332	36.4	242	33.9	308	29.5	−18.9	0.065
*P* value for heterogeneity	<0.001		<0.001		<0.001			
Maternal age (years)								
13–19	515	27.6	445	23.1	460	17.8	−35.5	<0.001
20–24	716	30.7	637	29.0	702	24.6	−19.9	0.011
25–29	622	25.2	614	28.0	638	24.0	−4.8	0.601
30 or more	687	27.7	683	24.3	853	21.9	−21.0	0.009
*P* value for heterogeneity	0.166		0.069		0.035			

^∗^Linearity deviation (*P* value for heterogeneity).

**Table 3:** Prevalence of smoking during pregnancy by family income, level of education, skin color, and age. Rio Grande, RS, Brazil, 2007–2013 (*N* = 7,572).

Variable/survey year	2007		2010		2013		% change	*P* value for trend in the study period
	*n*	%	*n*	%	*n*	%	2007–2013	
Overall	2,540	22.0	2.379	21	2.653	18.0	−18.2	<0.001
Family income quintile								
1 (lowest)	580	34.1	539	28.8	622	28.3	−17.0	0.029
2	449	26.1	473	30.4	504	21.8	−16.5	0.115
3	499	21.0	435	20.7	469	17.3	−17.6	0.143
4	526	17.1	513	15.6	533	14.3	−16.4	0.202
5 (highest)	486	10.3	419	6.9	525	6.7	−34.9	0.035
*P* value for linear trend	<0.001		<0.001^∗^		<0.001			
Level of education (full years)								
0–4	320	34.4	190	37.4	162	35.2	+2.3	0.769
5–8	917	32.1	884	31.8	892	29.3	−8.7	0.199
9–11	1,064	13.7	1,060	12.4	1,188	11.9	−13.1	0.189
12 or more	239	4.2	245	5.7	411	4.6	+9.5	0.889
*P* value for heterogeneity	<0.001		<0.001		<0.001			
Self-referred skin color								
White	1,671	19.5	1,630	16.9	1,751	15.3	−21.5	0.001
Mixed	517	24.9	507	29.8	593	22.3	−10.4	0.259
Black	332	29.2	242	29.3	308	25.0	−14.4	0.239
*P* value for heterogeneity	<0.001		<0.001		<0.001			
Maternal age (years)								
13–19	515	19.4	445	18.6	460	13.7	−29.4	0.020
20–24	716	24.4	637	22.6	702	19.4	−20.5	0.022
25–29	622	20.3	614	23.0	638	19.9	−2.0	0.870
30 or more	687	23.1	683	18.9	853	17.8	−22.9	0.010
*P* value for heterogeneity	0.107		0.125		0.041			

^∗^Linearity deviation (*P* value for heterogeneity).

**Table 4:** Prevalence of smoking cessation during pregnancy by family income, level of education, skin color, and age. Rio Grande, RS, Brazil, 2007–2013 (*N* = 1,930).

Variable/year of survey	2007		2010		2013		% change	*P* value for trend in the study period
	*n*	%	*n*	%	*n*	%	2007–2013	
Overall	709	18.0	626	21.1	595	17.6	−2.2	0.919
Family income quintiles								
1 (lowest)	242	14.9	173	11.6	210	12.4	−16.8	0.418
2	139	12.2	166	13.2	135	17.8	+45.9	0.192
3	139	23.7	123	26.8	103	20.4	−13.9	0.604
4	121	21.5	112	28.6	92	15.2	−29.3	0.072^∗^
5 (highest)	68	23.5	52	48.1	55	36.4	+54.9	0.020^∗^
*P* value for linear trend	0.012		<0.001		0.001			
Level of education (years)								
0–4	126	8.7	83	14.5	63	9.5	+9.2	0.674
5–8	349	13.7	336	16.7	309	14.2	+3.6	0.830
9–11	215	28.4	181	28.7	197	23.3	−17.9	0.258
12 or more	19	42.1	26	46.1	26	34.6	−17.8	0.573
*P* value for linear trend	<0.001		<0.001		<0.001			
Self-referred skin color								
White	412	17.7	368	25.8	336	19.3	+9.0	0.015^∗^
Mixed	165	18.2	176	14.2	167	16.8	−7.7	0.730
Black	121	19.0	82	14.6	91	13.2	−30.5	0.242
*P* value for heterogeneity	0.948		0.002		0.369			
Maternal age (years)								
13–19	142	23.2	103	20.4	82	20.7	−10.8	0.622
20–24	220	18.6	185	22.2	173	16.8	−9.7	0.702
25–29	157	15.9	172	18.0	153	16.3	+2.5	0.919
30 or more	190	15.3	166	23.5	187	18.2	+18.9	0.464
*P* value for linear trend	0.056		0.747		0.795			

^∗^Linearity deviation (*P* value for heterogeneity).
